# Knockdown of MRPL42 suppresses glioma cell proliferation by inducing cell cycle arrest and apoptosis

**DOI:** 10.1042/BSR20171456

**Published:** 2018-04-27

**Authors:** Chunyan Hao, Hubin Duan, Hao Li, Huan Wang, Yueting Liu, Yimin Fan, Ce Zhang

**Affiliations:** 1 Department of Geriatrics, First Clinical Medical College of Shanxi Medical University, 85 Jie Fang South Road, Taiyuan, 030001, Shanxi Province, People’s Republic of China; 2 Department of Neurosurgery, First Clinical Medical College of Shanxi Medical University, 85 Jie Fang South Road, Taiyuan, 030001, Shanxi Province, People’s Republic of China; 3 Department of Neurobiology, Shanxi Medical University, #56 Xin Jian South Road, Taiyuan 030001, Shanxi Province, People’s Republic of China

**Keywords:** apoptosis, cell cycle, glioma, MRPL42, proliferation

## Abstract

Mammalian mitochondrial ribosomal proteins are functionally involved in protein synthesis in mitochondrion. Recently numerous studies have illuminated the role of mitochondrion in cancer development. However, the precise function of mitochondrial ribosomal protein L42 (MRPL42) remains unclear. Here in the present study, we identified MRPL42 as a novel oncogene in glioma. By analyzing the Cancer Genome Atlas (TCGA) database, we first found that MRPL42 was significantly up-regulated in glioma tissues compared with normal tissues. Functionally, we silenced MRPL42 in glioma cells and revealed that MRPL42 knockdown largely blunted the proliferation of U251 and A172 cells. Mechanistically, our results suggested that MRPL42 silencing resulted in increased distribution of cell cycle in G_1_ and G_2_/M phases, while the S-phase decreased. In addition, the apoptosis and caspase3/7 activity were both activated after MRPL42 knockdown. Taken together, MRPL42 is a novel oncogene in glioma and might help us develop promising targetted therapies for glioma patients.

## Introduction

Glioma is a common type of brain tumor and derives from glial cells [1]. The regular treatment for this disease includes surgery, radiation, and chemotherapy, while the prognosis is still unsatisfactory [2]. Currently, genetic studies have identified a large amount of potential oncogenes or tumor suppressors in glioma, such as cyclin dependent kinase inhibitor 2B (CDKN2B) and regulator of telomere elongation helicase 1 (RTEL1) [3], isocitarte dehydrogenase (NADP^+^) 1 cytosolic (IDH1) [4], telomerase reverse transcriptase (TERT) and telomerase RNA component (TERC) [5]. However, curable therapies against this lethal malignancy are still limited. Thus, identifying potential pathogenic targets might help us in exploring effective therapeutic treatment.

Mammalian mitochondrial ribosomal proteins are a cluster of factors that are encoded by nuclear genes and are responsible for protein synthesis in mitochondrion. Generally, mitochondrial ribosome are composed of a large 39S subunit and a small 28S subunit. Some studies have identified mitochondrial ribosomal proteins, death-associated protein 3 (DAP3) and mitochondrial ribosomal protein S30 (PDCD9) as apoptosis-triggering factors [6,7]. It has also been reported that X-linked ribosomal protein S4 high expression is correlated with slower ovarian tumors progression and better survival [8]. In addition, mitochondrial ribosomal protein L41 (MRPL41) has been demonstrated to inhibit the xenografted tumor growth of cancer cells through stabilizing P53 [9]. Another study has found that MRPL41 participates in cell-cycle arrest induced by serum starvation [10]. Similar to MRPL41, mitochondrial ribosomal protein L42 (MRPL42) is also a family member of mammalian mitochondrial ribosomal proteins. It belongs to 28S and 39S subunits and its role in glioma is undetermined.

We initially noticed that MRPL42 was obviously up-regulated in glioma tissues according to the The Cancer Genome Atlas (TCGA) database. Based on lentivirus-mediated knockdown strategy, we found that MRPL42 reduction largely suppressed the proliferation of U251 and A172 glioma cells. We also revealed that cell cycle was arrested as shown by the increased percentage in G_1_, G_2_/M and decreased distribution in S phases. Apoptosis and caspase-3/caspase-7 activity were enhanced in shMRPL42 glioma cells. These results indicated that MRPL42 knockdown suppressed the glioma cell viability at least partly through disturbing cell cycle progression and activating apoptosis.

## Materials and methods

### TCGA gene expression analysis

Transcriptional abundance of glioma-related MPRL42 was excavated from TCGA (http://cancergenome.nih.gov). In brief, a total of 558 glioma specimens and 10 normal tissues were available for the analysis.

### Cell culture

Normal human astrocytes (NHA) and human glioma cells U87, U373, U251, and A172 were obtained from American Type Culture Collection. All the cells were cultured in Dulbecco’s modified Eagle’s medium (Invitrogen), supplemented with 10% FBS (HyClone) and 1% penicillin and streptomycin solution (Corning). The cells were maintained in 37°C incubator with 5% CO_2_.

### Total mRNA isolation and quantitative real-time PCR

For total RNA isolation, indicated cells were lysed using TRIzol reagent (Invitrogen) and then subjected to RNeasy Mini kit (Qiagen). Reverse transcription was performed using ReverTra Ace® qPCR RT Master Mix with gDNA Remover (TOYOBO), following the manufacturer’s instructions. TransStart Top Green qPCR SuperMix (TransGen Biotech) was used for transcripts detection. Glyceraldehyde-3-phosphate dehydrogenase (GAPDH) serves as internal control. The real-time PCR primers were listed as followed: MPRL42 forward: and reverse GAPDH forward: 5′-TGACTTCAACAGCGACACCCA-3′ and reverse: 5′-CACCCTGTTGCTGTAGCCAAA-3. The relative expression of MPRL42 was normalized to GAPDH and analyzed using comparative Δ*C*_T_ method (*C*_T_^MRPL42^ − *C*_T_^GAPDH^). A lower *C*_T_ value represents a higher relative expression of MRPL42.

### Western blot

Indicated cells were lysed in lysis buffer (1 g SDS, 0.78 g DTT, 3 ml Tris (1 M, pH 6.8), 5 ml glycerol and ddH_2_O up to 50 ml). After incubation for 10 min at room temperature, cell lysis was boiled at 98°C for 10 min and centrifuged for 5 min. After running on SDS/PAGE (12% gel) for 1.5 h, the proteins were transferred on to PVDF membrane (Millipore) for 1 h and then blocked with 5% skim milk for 1 at room temperature. Then the proteins on PVDF membrane were incubated with primary antibodies at 4°C overnight and indicated secondary antibodies.

### MPRL42 knockdown in U251 and A172 cells

MPRL42 was knocked down using pGCSIL-GFP (stably expressed shRNA containing a GFP marker) lentivirus system. Briefly, shRNA against MPRL42 (5′-CAAAGAGAACTATCTTGAA-3′) or shCtrl (5′-TTCTCCGAACGTGTCACGT-3′) was inserted into pGCSIL-GFP vector by GeneChem (Shanghai, China). 293T cells were transfected with pGCSIL-GFP and packaging vectors (pHelper1.0: gag/pol and Helper2.0: VSVG) using Lipofectamine 2000 (Invitrogen), following the manufacturer’s instructions. Forty-eight hours after transfection, viral supernatants were collected and filtered through a 0.45-μm filter.

### High-content screening assay

Equal numbers of shCtrl and shMPRL42 U251 or A172 cells were seed in 96-well plates and maintained for 5 days. The stained cells were photographed using a fluorescence fluorescence-imaging microscope (20× objective). Cell numbers from days 1 to 5 was determined by the ArrayScan™ HCS software (Cellomics Inc).

### Cell proliferation analysis

U251 and A172 cells expressing shCtrl or shMPRL42 lentivirus were subjected to MTT assay for cell proliferation analysis. Briefly, a total of 3000 indicated cells were seeded in 96-well plates and cell viability was measured at day 1, 2, 3, 4, or 5. For clarity, MTT solution (5 mg/ml) was added into 96-plate wells and incubated at 37°C. Three hours later, cell culture and MTT solution were removed and 150 μl DMSO was added into the plates. The optical density (OD) was detected at 490 nm by a microplate reader.

### Cell cycle determination

Propidium iodide (PI) staining was performed to analyze the cell cycle. shCtrl or shMPRL42 U251 and A172 cells were seeded in six-well plates. PI absorbance was determined on a flow cytometer.

### Apoptosis assay

Cell apoptosis was detected using annexin V-APC apoptosis detection kit (Ebioscience), following the manufacturer’s protocol. shCtrl or shMPRL42 U251 and A172 cells were washed with PBS, and then resuspended in staining buffer at a density of 1 × 10^6^ ml. Five microliters of annexin V-APC was added and the mixture was maintained at room temperature for 15 min, then subjected to flow cytometry analysis (FACSCalibur, Becton-Dickinson).

### Caspase 3/7 activity measurement

Ten thousand indicated cells were seeded into 96-well plates, and then 100 μl caspase-Glo reagent (Promega) was added into the plates according to the manufacturer’s protocol. The plates were rotated at 400 rpm for 30 min and incubated at room temperature for 90 min. The activity was determined on a microplate reader.

### Statistical analysis

All the statistical data were analyzed in at least three independent experiments. Unpaired Student’s *t* test was used to analyze the difference between two groups. Difference was determined by one-way ANOVA in more than two groups. All the statistical analyses were performed using GraphPad Prism 6 software. *P*-value less than 0.05 was considered significant.

## Results

### MRPL42 level is increased in glioma specimens

**Figure 1:**
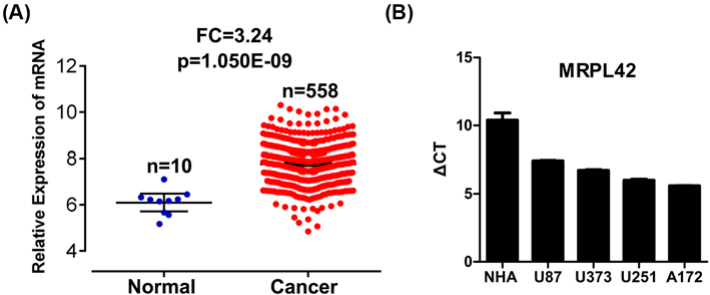
MRPL42 is up-regulated in glioma specimens (**A**) Relative mRNA expression of MRPL42 in glioma and normal tissues that are analyzed from TCGA database (fold change = 3.24, *P*=1.050E-09). Normal tissues, *n*=10; glioma tissues, *n*=558. (**B**) The mRNA abundance was determined by qRT-PCR assay in NHA and four glioma cell lines U87, U373, U251, and A172. GAPDH serves as the internal control. The relative expression of MRPL42 is shown as Δ*C*_T_ (*C*_T_^MRPL42^ − *C*_T_^GAPDH^). A lower *C*_T_ value represents a higher relative expression of MRPL42.

By analyzing the RNA sequencing data from the TCGA database, we found that MRPL42 abundance was obviously increased in a total of 558 glioma tissues as compared with ten normal tissues (Figure 1A) (fold change = 3.24, *P*=1.050E-09). We also detected the mRNA level of MRPL42 using quantitative real-time PCR (qRT-PCR) in NHA and four glioma cell lines U87, U373, U251, and A172. The results showed that MRPL42 level was higher in U87, U373, U251, and A172 compared with NHA (Figure 1B).

### MRPL42 knockdown suppresses the proliferation of U251 and A172 cells

**Figure 2:**
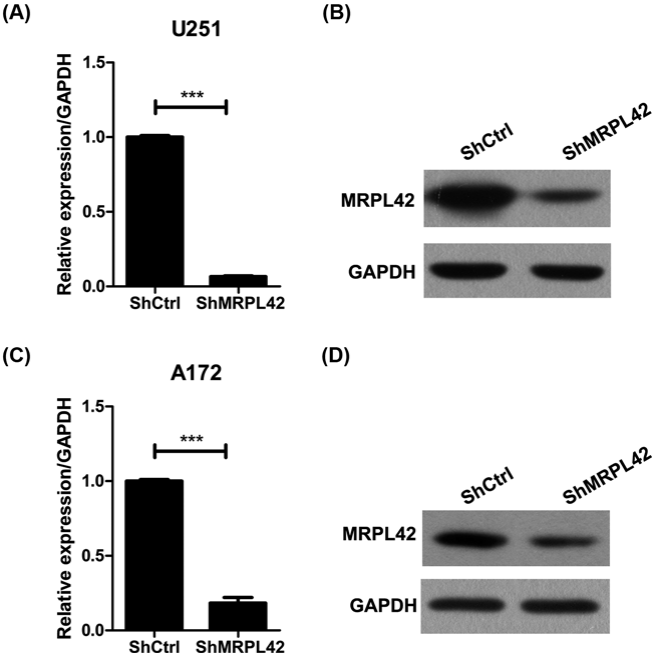
MRPL42 is efficiently knocked down in U251 and A172 cells using lentivirus silencing strategy (**A**,**B**) qRT-PCR (A) and Western blot (B) analysis of MRPL42 in U251 cells expressing shCtrl or shMRPL42 lentivirus; ***P<0.001. (**C**,**D**) qRT-PCR (C) and Western blot (D) analysis of MRPL42 in A172 cells expressing shCtrl or shMRPL42 lentivirus; ***P<0.001.

**Figure 3:**
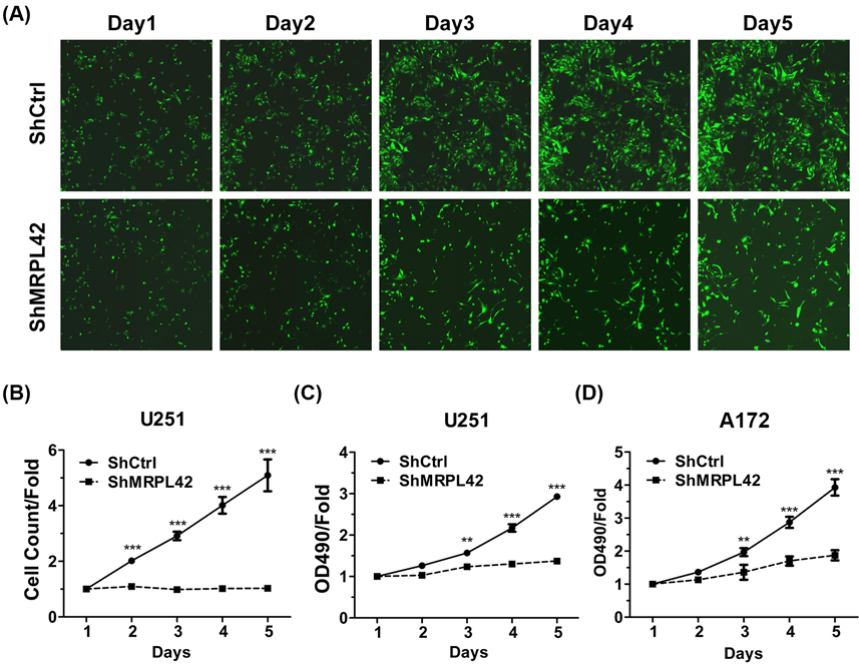
MRPL42 silencing suppresses the proliferation of U251 and A172 cells (**A**,**B**) Cell viability of shCtrl and shMRPL42 U251 cells were analyzed using multiparametric HCS assay from days 1 to 5. (A) Representative photographs of HCS. (B) Quantitative results of HCS. ***P<0.001. (**C**) shCtrl and shMRPL42 U251 cells were subjected to MTT analysis of cell proliferation at the indicated time. **P<0.01, ***P<0.001. (**D**) shCtrl and shMRPL42 A172 cells were subjected to MTT analysis of cell proliferation at the indicated time. **P<0.01, ***P<0.001.

To investigate the role of MRPL42 in glioma, we knocked down MRPL42 using lentivirus shRNA strategy in U251 and A172 cells and examined the cell viability. First, we found that MRPL42 was efficiently silenced in shMRPL42 U251 or A172 cells as shown by the qRT-PCR and Western blot results (Figure 2). Next, we detected cell growth using high-content screening (HCS) assay at 1, 2, 3, 4, and 5 days after cell seeding. The fluorescence images of the cells suggested that the viability of shMRPL42 U251 cells was constantly lower than that of shCtrl U251 cells (Figure 3A). The shCtrl U251 cells grew intensely from days 1 to 5, while the shMRPL42 U251 cells had limited proliferative potential (Figure 3A). We also quantitated the relative cell numbers and the results showed that MRPL42 knockdown largely blunted the proliferation of U251 cells (Figure 3B). We further performed MTT assay to analyze the proliferation rate and the results were consistent to the HCS analysis (Figure 3C). To explore whether this suppressive effect is limited to U251 cells, we silenced MRPL42 in another glioma cells A172. Likewise, based on MTT assay, we also showed that MRPL42 silencing inhibited the proliferation of A172 cells (Figure 3D). In conclusion, our findings revealed that MRPL42 was critical for glioma cell proliferation.

### MRPL42 knockdown induces the cell cycle arrest of U251 and A172 cells

**Figure 4:**
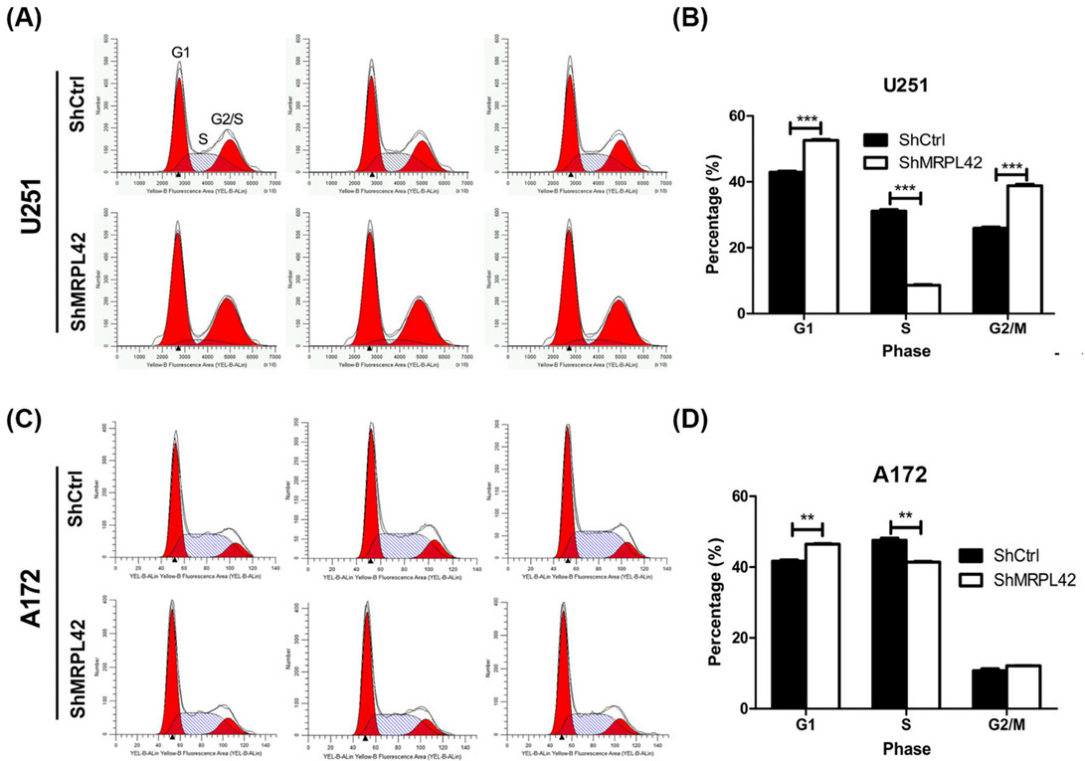
MRPL42 knockdown leads to cell cycle arrest of U251 and A172 cells (**A**) Cell cycle of U251 cells was analyzed using flow cytometry 72 h after shCtrl or shMRPL42 lentivirus infection. Left, G_1_ phase; Middle, S phase; Right, G_2_/M phase. (**B**) Quantitative analysis of G_1_, S, and G_2_/M phase of U251 cells expressing shCtrl or shMRPL42 lentivirus. ***P<0.001. Quantitation was determined by the area under the indicated phase. (**C**) Cell cycle of A172 cells was analyzed using flow cytometry 72 h after shCtrl or shMRPL42 lentivirus infection. (**D**) Quantitative analysis of G_1_, S, and G_2_/M phase of A172 cells expressing shCtrl or shMRPL42 lentivirus. ** *P*<0.01.

Because cell cycle is the primary event of cell proliferation, we first focussed on whether MPRL42 regulated the progression of cell cycle. PI staining detected by flow cytometer was performed in shCtrl and shMPRL42 U251 and A172 cells. Our results showed that MPRL42 knockdown resulted in increased distribution in G_1_ and G_2_/M phases and decreased distribution in S-phase (Figure 4). This indicates that MPRL42 knockdown led to cell cycle arrest at the G_1_ and G_2_/M phases.

### MRPL42 knockdown activates the apoptosis of U251 and A172 cells

**Figure 5:**
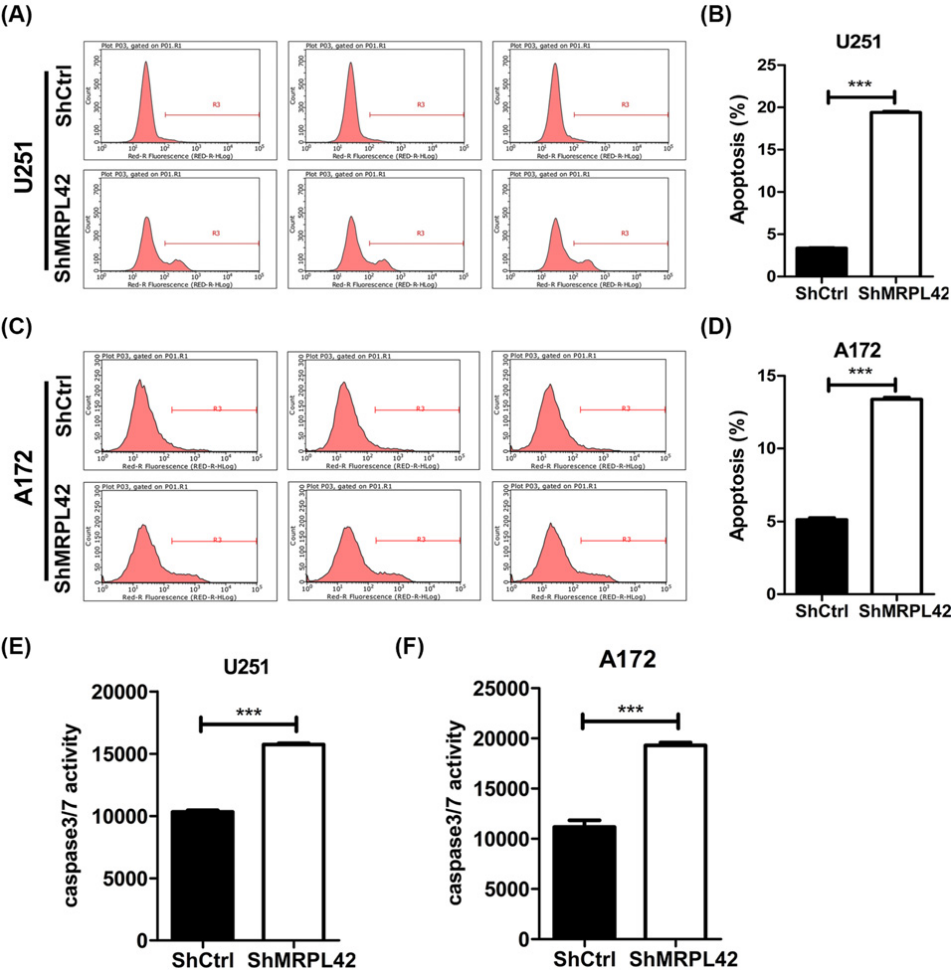
MRPL42 knockdown induces U251 and A172 cells apoptosis (**A**) Cell apoptosis of U251 cells was determined by Annexin V-APC staining and flow cytometry 72 h after shCtrl or shMRPL42 lentivirus infection. Red-R fluorescence >10^2^ represented apoptotic cell area. (**B**) Quantitative result of apoptosis as shown in (A), ***P<0.001. (**C**) Cell apoptosis of A172 cells was determined by Annexin V-APC staining and flow cytometry 72 h after shCtrl or shMRPL42 lentivirus infection. Red-R fluorescence >10^2^ represented apoptotic cell area. (**D**) Quantitative result of apoptosis, ***P<0.001. (**E**) shCtrl and shMRPL42 U251 cells were subjected to caspase 3/7 activity determination. ***P<0.001. (**F**) shCtrl and shMRPL42 A172 cells were subjected to caspase 3/7 activity determination. ***P<0.001.

Decreased cell proliferation induced by MRPL42 knockdown may be a consequence of increased cell death. Thus, we analyzed the apoptosis in shCtrl and shMPRL42 U251 and A172 cells. We found that shMPRL42 U251 cells had higher apoptotic cell number compared with that of shCtrl U251 cells (Figure 5A,B). We also checked the caspase activity and found that caspase-3/caspase-7 activity was increased after MPRL42 knockdown in U251 cells (Figure 5E). Consistently, the apoptosis (Figure 5C,D) and caspase-3/caspase-7 (Figure 5F) activity were both enhanced in shMPRL42 A172 cells compared with shCtrl A172 cells.

## Discussion

In the present study, we demonstrated that MRPL42 was critical for glioma cell survival. We observed an up-regulation of MRPL42 in glioma tissues by analyzing the TCGA database. Thus, we checked MRPL42 abundance in glioma cells and found that the mRNA level of MRPL42 was higher in glioma cells U87, U373, U251, and A172 compared with NHA cells. These evidences might suggest an essential role of MRPL42 for glioma progression. *In vitro*, we knocked down MRPL42 in glioma cells and the results showed that MRPL42 silencing largely blunted the proliferation of U251 and A172 cells, indicating that glioma cell survival was depending on MRPL42 expression. In addition, cell cycle arrest and apoptosis were induced after MRPL42 knockdown, which could partly explain the reduced cell viability of MRPL42 silencing U251 and A172 cells.

Mitochondria is a well-known energy factory involved in oxidative phosphorylation, fatty acid oxidation, and the synthesis of lipids, nucleotides, and amino acids [11]. In the past decade, studies have revealed potential correlation of mitoribosome proteins in cancer biology. *MRPL37* mRNA expression is increased in lymphoma human tissues and cells [12]. However, some cancer types and cells exhibit decreased level of MRPL41 [9]. Although some studies have detected dysregulation of mitoribosome proteins in cancers, the precise role is still undetermined. MRPL42 is also a mitoribosome protein that its gene is located on chromosome 12 [13]. In this study, we investigated the function of MRPL42 in glioma and found that its mRNA level was evaluated in glioma tissues. Functional study using lentivirus knockdown strategy helped us illustrate the important role of MRPL42 in glioma that MRPL42 silencing resulted in suppressed growth of glioma cells. This implied that this protein was necessary for glioma cell survival. It is known that mitochondria proteins are closely correlated with programmed cell death or apoptosis [14]. It has been reported that MPRL42 binds to Bcl-2 to induce apoptosis [15]. MPRL42, also known as programmed cell death protein 9, is homologous to chicken p53 protein. Overexpression of MPRL42 leads to up-regulation of c-jun and activation of JNK signaling, and subsequently enhanced apoptosis [16]. Here, apoptosis was activated in MPRL42 glioma cells. Additionally, caspase-3/caspase-7 activity was also increased after MPRL42 knockdown, suggesting that MPRL42 plays important roles in apoptosis. However, how MPRL42 regulates apoptosis is still unknown. Future studies are needed to clarify the molecular mechanisms of MPRL42 knockdown induction of apoptosis. Our findings also revealed that MPRL42 knockdown glioma cells had a lower distribution of S-phase and increased percentage of G_1_ and G_2_/M phases. This implied that shMPRL42 glioma cells were retarded at the G_1_ and G_2_/M phases, indicating enhanced cell cycle arrest.

In summary, we provide for the first time that MPRL42 functions as an oncogene in glioma. Up-regulation of MPRL42 is observed in glioma tissues and cells. Knockdown of MPRL42 largely suppresses the proliferation and growth of glioma cells. Mechanistically, enhanced cell cycle arrest at the G_1_ and G_2_/M phases and increased apoptosis were found in MPRL42 silencing glioma cells.

## Abbreviations

GAPDH: glyceraldehyde-3-phosphate dehydrogenase
HCS: high-content screening
MRPL41: mitochondrial ribosomal protein L41
MRPL42: mitochondrial ribosomal protein L42
NHA: normal human astrocyte
PI: propidium iodide
qRT-PCR: quantitative real-time PCR
TCGA: The Cancer Genome Atlas
