# Expression of Concern: Knockdown of MRPL42 suppresses glioma cell proliferation by inducing cell cycle arrest and apoptosis

**DOI:** 10.1042/BSR20171456_EOC

**Published:** 2026-07-24

**Authors:** Chunyan Hao, Hubin Duan, Hao Li, Huan Wang, Yueting Liu, Yimin Fan, Ce Zhang

**Affiliations:** 1 Department of Geriatrics, First Clinical Medical College of Shanxi Medical University, 85 Jie Fang South Road, Taiyuan, 030001, Shanxi Province, People's Republic of China; 2 Department of Neurosurgery, First Clinical Medical College of Shanxi Medical University, 85 Jie Fang South Road, Taiyuan, 030001, Shanxi Province, People's Republic of China; 3 Department of Neurobiology, Shanxi Medical University, #56 Xin Jian South Road, Taiyuan 030001, Shanxi Province, People's Republic of China

**Keywords:** apoptosis, cell cycle, glioma, MRPL42, proliferation

The Editorial Office has been made aware of potential issues surrounding the scientific validity of this paper and is hence issuing a permanent expression of concern to notify readers.

Unexpected similarities have been noted between the MRPL42 bands presented in Figure 2B of the manuscript and the Caspase-3 band presented in Wang et al. 2018 (DOI: 10.1042/BSR20180574 [retracted]) following a vertical flip. The authors have been contacted for comment and have not responded or provided the requested raw data.

As the issues are unable to be resolved, this expression will remain published permanently or until further evidence arises.

